# Medical Students’ Preferences of Study Resources: Physical vs Digital Resources

**DOI:** 10.7759/cureus.56196

**Published:** 2024-03-14

**Authors:** Marwah Al Shmanee, Moaaz Issa, Hind Alkholy, Amna Alnaqbi, Abdalrhman Awadallah, Hadil Hassan, Amal Hussein

**Affiliations:** 1 College of Medicine, University of Sharjah, Sharjah, ARE; 2 Department of Family and Community Medicine and Behavioral Sciences, University of Sharjah, Sharjah, ARE

**Keywords:** medical education, influencing factors, physical vs digital resources, digital resources, physical resources, medical students' resource preferences, study resources

## Abstract

Introduction

Medical school students are presently confronted with distinct study resources, that can be categorized into physical and digital formats. Additionally, existing literature offers limited insights into the most efficacious and favored modalities of study adopted by medical students. The following research seeks to elucidate the optimum mode of study embraced by medical students, concurrently examining the determinants influencing their choices.

Methods

A cross-sectional study was conducted, encompassing a cohort of 572 students from the Colleges of Medicine and Dental Medicine at the University of Sharjah, Sharjah, UAE. All the students in the target population were invited to participate in this study by completing a self-administered questionnaire. The ensuing data analysis was executed using SPSS version 25 (IBM Corp., Armonk, NY).

Results

Among the participants, 184/570 individuals were male, constituting 32.3% of the total sample, while 386/570 participants were female, representing 67.7% of the sample. A predominant proportion of students 355/567 (62.6%) articulated a preference for both modalities of studying, whereas those exclusively favoring either physical or digital formats constituted 119/567 (21%) and 93/567 (16.4%), respectively. Notably, an association was discerned between both colleges and the favored mode of study, indicating that a higher percentage of medical students 198 (53.8%) exhibited a preference for recommended textbooks compared to their counterparts in dental medicine 60 (29.4%) (P-value <0.001). The utilization of printed textbooks demonstrated a decline among third-year students 66 (35.7%) when juxtaposed with first and second-year students 97 (49.5%) and 94 (49.7%), respectively (P-value = 0.001). The prevailing inclination among students was to select learning resources based on the quality of information provided 457/571 (80%) and considerations pertinent to their time and schedule 501/572 (87.6%).

Conclusion

Students within the colleges of medicine and dental medicine enhance their educational experiences through the utilization of a variety of learning resources encompassing both physical and digital modalities. Among the factors influencing their selection, only the quality of information proffered by the learning source and schedule held substantive significance.

## Introduction

With the rapid technological advancements in the 21st century, medical students are continually faced with newly emerging healthcare knowledge that they are expected to know. Even though this study was conducted before the transformative global impact of the COVID-19 pandemic and before the current easy access to artificial intelligence, the new medical information and study resources being readily available for medical students with all the scientific evolution puts them in great confusion [1].

Medical resources are typically classified as physical and digital resources, with physical being defined as traditional resources such as textbooks and any other tangible resources [2]; digital resources are any resources on the web that are easily accessible wherever you are [3]. Both resources are different, yet beneficial.

Students who prefer using physical resources, particularly textbooks, as their primary studying source find them better than other sources because textbooks are scientifically approved, checked, and better for research and individual study. Textbooks also provide more organized information and are rich with details [4]. Moreover, students comprehend more when they write their notes by hand, interact with concrete experiences that stimulate tactile reactions, and simply traverse printed materials while keeping the larger text frame visible, unlike digital resources [5].

On the other hand, digital resources are easier to find and use around the clock. It is easy to navigate through them and retrieve the pertinent information. Digital resources also provide hyperlinks to sources with additional information about a particular topic [6]. In addition, the increasing availability of online resources, along with students' acquisition and possession of electronic devices, leads to a pronounced preference for digital resources. This inclination is mainly attributed to the portability of digital resources, allowing students to carry multiple resources simultaneously, an advantage that is not offered by physical resources [5].

Students in their initial years of medical school, known as preclinical students, are those whose medical curriculum focuses primarily on basic biosciences, in which a major source of their medical knowledge is acquired from lectures and lecture notes. However, because students are confronted by an abundance of information and constrained by little available time, they tend to resort to gaining superficial knowledge by scanning through many resources. Thus, they rely heavily on themselves to decide which resources to study from [7-9].

Literature that enumerates the types of study resources used by medical students globally is currently limited. Previous studies on the educational resources used by medical students primarily demonstrate their preferences during a primary healthcare rotation [10,11]. Other research on medical students' resource use and behavior in selecting specific resources is available; however, these studies were mainly conducted in Canada [12], the United States [6], Australia [2,7], the United Kingdom [11], and the Netherlands [13]. To our knowledge, a single study was carried out in the Middle East region, specifically in Saudi Arabia. The participants of this study favored the use of online resources such as general websites, medical websites, digital textbooks, and online journals. It also demonstrated that most of the students’ study time is spent on lecture notes and handouts [14]. With this in mind, no studies were found to be conducted in the United Arab Emirates, implying a lack of local information displaying the student’s preferred form of studying resources and the influencing factors.

Given that medical students are now born and raised in a technologically advancing world [5] and because some studies claim that books become obsolete prior to their publication [1], we hypothesize that students would prefer the usage of digital resources as the preferred form of studying resources, as compared to physical resources. Thus, the study aims to explore the various learning resources used by pre-clinical medical students in a private university located in the United Arab Emirates (UAE), determine whether physical or digital resources are preferred, and identify factors influencing students’ preferences. The findings of this study would help in providing valuable insights about the different studying resources used by medical students, assisting them in selecting the most effective options for their learning needs. Furthermore, these insights would aid medical education tutors and university professors in understanding the studying habits of medical students and facilitating the development of tailored strategies for the continuous improvement of medical education practices. The findings of this research were also presented at the Towards Unity for Health (TUFH) 2023 “Beyond Boundaries: Health Equity through a Culture of Learning” conference, co-hosted by The Network: TUFH and the University of Sharjah, which was conducted at the University of Sharjah, Sharjah, UAE, during the period from October 23 to 26, 2023.

## Materials and methods

Study design

This is a quantitative cross-sectional study that was conducted between January 28 and February 17, 2020. The target population comprised all medical students in the pre-clinical phase within the faculties of medicine and dental medicine at the University of Sharjah, Sharjah, UAE. Notably, individuals classified within the pre-clinical years encompass those in their initial, second, and third years engaged in foundational scientific studies. This research primarily concentrates on preclinical medical students, given the notable distinctions in studying approaches between preclinical and clinical students. The intention is to lay the groundwork for future research specifically targeting the study habits of clinical students.

Sampling method (inclusion and exclusion criteria)

Participants satisfied the study’s inclusion criteria if they were (1) medicine and dental medicine students, (2) in the pre-clinical years, (3) available at the time of data collection, and (4) agreeing to participate in this study. Individuals who failed to fulfill our inclusion criteria were excluded from the study. The minimum sample size needed to conduct this study was 385, which was calculated using Cochrane’s formula for sample size calculation in a cross-sectional study: n = Z_a/2_^2^ * p * (1-p) / ME^2^; where Z_a/2_ was set at 1.96, corresponding to a type I error of 5%, the expected prevalence (p) was set at 50%, and the margin of error (ME) was set at 5%. In the context of the sampling method, our research team aimed to include all the target populations in the study. To accomplish this, we compiled a comprehensive list of all academic lectures conducted in both colleges, namely the College of Medicine and Dental Medicine. Invitations were extended to students from both colleges during core lectures, where attendance is mandatory. The utilization of an attendance sheet further ensured that all students present during the data collection period were invited to participate in our study. This meticulous approach helped mitigate the risk of overlooking any individual during the data collection phase. 

Tools of data collection

Data collection was performed during the beginning of the second semester of the academic year. Printed questionnaires were distributed to all the study participants, who were asked to fill out the questionnaires by themselves. The questionnaire consisted of 38 questions, which were divided into three main sections. Section (A) Demographics, Section (B) Resources used by medical students, and Section (C) Influencing factors. All the questions were constructed by the researchers based on a thorough revision of existing literature. The questions varied in type, with closed-ended, multiple-choice questions making up most of the questionnaire. A 5-point Likert scale was used to assess the frequency of using both forms of study resources, ranging from never to every time, as well as agreement with several statements concerning factors that influence students' choice of study resources, ranging from strongly agree to strongly disagree. The questionnaire was pilot-tested among a sample of 30 students. The content validity of the questionnaire was reviewed and assured by two senior research supervisors who are experts in the field of medical education research, and modifications were made based on their insightful suggestions.

Data analysis

Data entry, coding, and analysis were conducted using the Statistical Package for Social Sciences (SPSS version 25) software (IBM Corp., Armonk, NY) [15]. Descriptive statistics, including frequencies and percentages, were used to summarize categorical variables while means and standard deviations (SD) were reported for scale variables. Chi-square tests were used to measure the association between two categorical variables. A P-value less than 0.05 was considered statistically significant. Bar and pie charts were used to visually demonstrate the results of this study. Total responses for each variable were demonstrated by using the valid total, which excludes missing data and accounts for valid cases only. Percentages were reported as valid percentages for all variables.

Ethical approval

This research has been approved by the University of Sharjah Research Ethics Committee (reference number: REC-20-01-27-07-S). Before distributing the questionnaire, all medical students were informed of the study’s purpose, and their verbal consent was first obtained. An information sheet for participation in the research study was included with the survey, and it included details about the study's objectives, participants' rights, and the duration of time needed to complete the questionnaire. Confidentiality was ensured by storing the data in password-protected electronic devices with strict access provided solely to the researchers. The study participants were not offered any incentives or compensations for their participation in the study.

## Results

Demographics

This study targeted a total of 764 pre-clinical students enrolled at the Colleges of Medicine and Dental Medicine at the University of Sharjah, Sharjah, UAE. Data were collected from 577 students who consented to participate in the study. Out of these respondents, five were excluded from the study due to their incomplete data. As a result, 572 participants were included in the study with a response rate of 572/764 (74.9%). The total number of participants and the percentages used in the upcoming results are valid totals and valid percentages, which exclude missing data and account only for valid responses. 

Among the study participants, there were 184/570 (32.3%) males and 386/570 (67.7%) females. Participants’ ages ranged between 18 and 24 years with a mean age of 19.95 years (SD = 1.109). Of all respondents, 204/572 (35.7%) were from the College of Dental Medicine, while 368/572 (64.3%) were from the College of Medicine. The distribution of respondents across the three preclinical years in the College of Medicine was uniform with an average response of 21.5%. Similarly, 11.8% of respondents from the College of Dental Medicine were from each of the three preclinical years. Family income less than 16,499 AED was reported by 114/513 (22.2%) of students, between 16,500 AED and 27,499 AED by 159/513 (31.0%), while family income greater than 27,500 AED was reported by 240/513 (46.8%) of students. Table 1 summarizes the demographic characteristics of study participants.

**Table 1:** The sociodemographic characteristics of medical students who participated in our study *The valid total excludes missing data and accounts only for valid responses

Demographic groups	Subgroups	Frequency	Valid Percent (%)
Gender	Male	184	32.3
	Female	386	67.7
	*Valid Total	570	
Age (in years)	18	45	8.1
	19	156	28.1
	20	191	34.4
	21	114	20.5
	22	43	7.7
	23	5	0.9
	24	1	0.2
	*Valid Total	555	
College	College of Medicine	368	64.3
	College of Dental Medicine	204	35.7
	*Valid Total	572	
Current academic year per college	Medicine year 1	130	22.8
	Medicine year 2	121	21.2
	Medicine year 3	117	20.5
	Dental Medicine year 1	66	11.6
	Dental Medicine year 2	68	11.9
	Dental Medicine year 3	68	11.9
	*Valid Total	570	
Total family income	Less than 16,499 AED	114	22.2
	16,500 AED – 27,499 AED	159	31.0
	More than 27,500 AED	240	46.8
	*Valid Total	513	

Preferences of study resources

**Figure 1:**
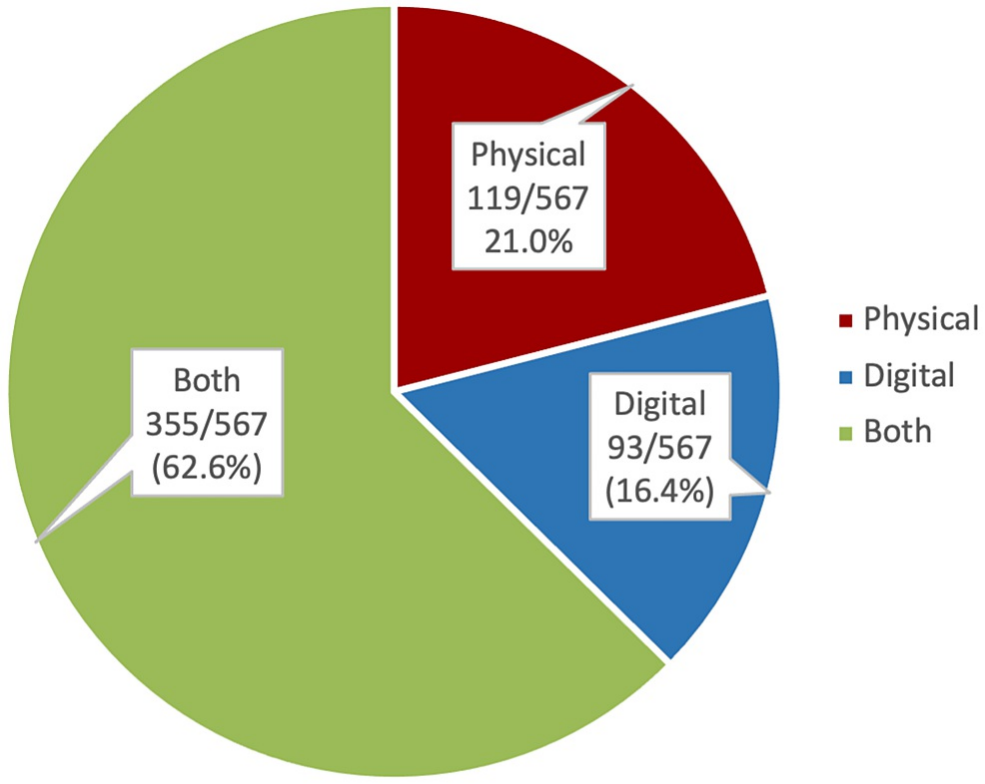
Medical students preferred form of study resources The data are represented above as frequency/valid total (valid percent). A valid total excludes missing data and accounts only for valid responses.

**Figure 2:**
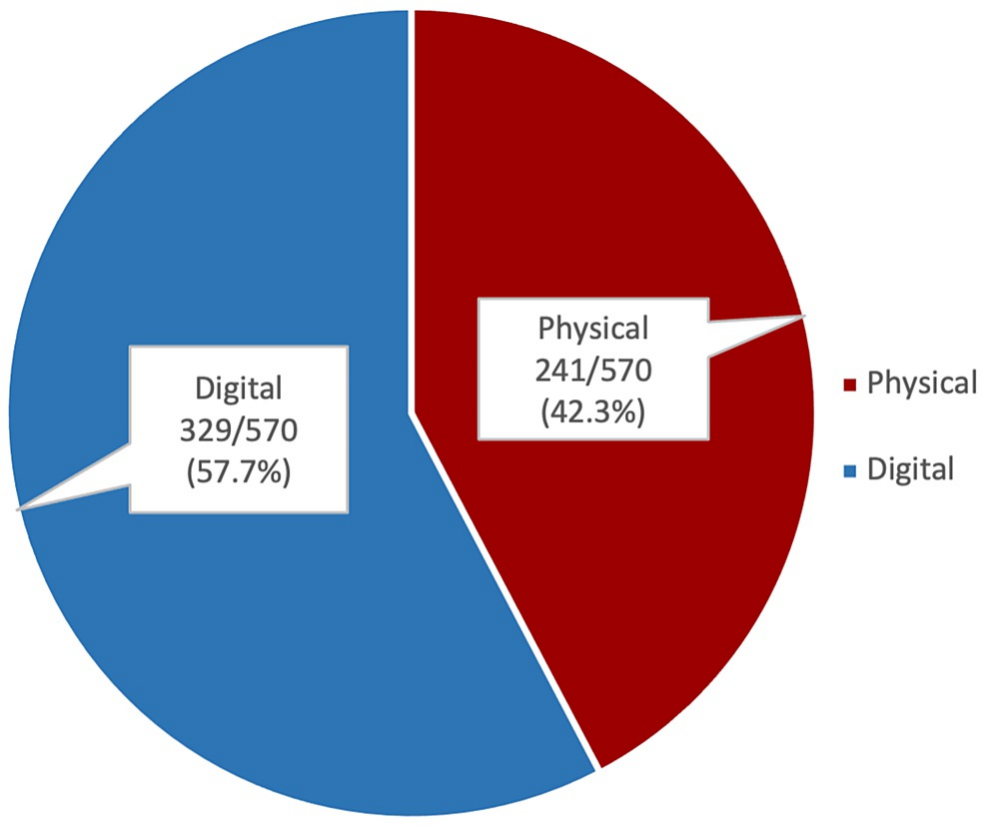
Preferred form of study resources as a last-minute revision before an exam The data are represented above as frequency/valid total (valid percent). A valid total excludes missing data and accounts only for valid responses.

The majority of students 355/567 (62.6%) expressed a preference for utilizing both physical and digital forms of study resources, while those who preferred using solely physical or digital forms were 119/567 (21.0%) and 93/567 (16.4%), respectively (Figure 1). However, more than half of the students 329/570 (57.7%) were likely to use a digital form of study resource as a last-minute revision before an exam, as compared to 241/570 (42.3%) who would prefer a physical resource (Figure 2).

Utilization of study resources

The most commonly used physical study resource was printed lecture handouts which were used every time or almost every time by 355/572 (62%) of students. In comparison, digital lecture handouts were the most commonly used digital study resource every time or almost every time by 360/567 (63.5%) of students. This is followed by YouTube videos as the second most used digital resource by 275/568 (48.4%) of students every time and almost every time. Random search engines and online website platforms took third and fourth place among the most used digital study resources every time or almost every time with almost equal usage of 225/565 (39.9%) and 224/567 (39.5%) respectively (Table 2).

**Table 2:** Students' level of usage of physical and digital resources *The valid total excludes missing data and accounts only for valid responses

Study resource	*Valid Total Responses	Never n (valid %)	Almost Never n (valid %)	Occasionally/ Sometimes n (valid %)	Almost every time n (valid %)	Every time n (valid %)
Physical Resources						
Recommended Textbooks	572	86 (15.0%)	115 (20.1)	258 (45.1)	85 (14.9)	28 (4.9)
Printed Journals/Articles	571	171 (29.9)	218 (38.2)	152 (26.6)	16 (2.8)	14 (2.5)
Printed Lecture Handout	572	55 (9.6)	46 (8.0)	116 (20.3)	86 (15.0)	269 (47.0)
Digital Resources						
YouTube Videos	568	20 (3.5)	32 (5.6)	241 (42.4)	153 (26.9)	122 (21.5)
Online Website Platforms ex. Lecturio, Amboss, Sketchy and others	567	57 (10.1)	83 (14.6)	203 (35.8)	143 (25.2)	81 (14.3)
Online Articles	568	137 (24.1)	189 (33.3)	175 (30.8)	37 (6.5)	30 (5.3)
Wikipedia	567	137 (24.2)	97 (17.1)	194 (34.2)	87 (15.3)	52 (9.2)
Random Internet Search Engines	565	68 (12.0)	82 (14.5)	190 (33.6)	136 (24.1)	89 (15.8)
Facebook	567	398 (70.2)	96 (16.9)	45 (7.9)	14 (2.5)	14 (2.5)
Twitter	566	372 (65.7)	70 (12.4)	59 (10.4)	33 (5.8)	32 (5.7)
WhatsApp	565	185 (32.7)	86 (15.2)	107 (18.9)	81 (14.3)	106 (18.8)
Educational Applications	567	100 (17.6)	99 (17.5)	210 (37.0)	95 (16.8)	63 (11.1)
Digital Lecture Handouts	567	37 (6.5)	43 (7.6)	127 (22.4)	115 (20.3)	245 (43.2)

Source of instructions

**Figure 3:**
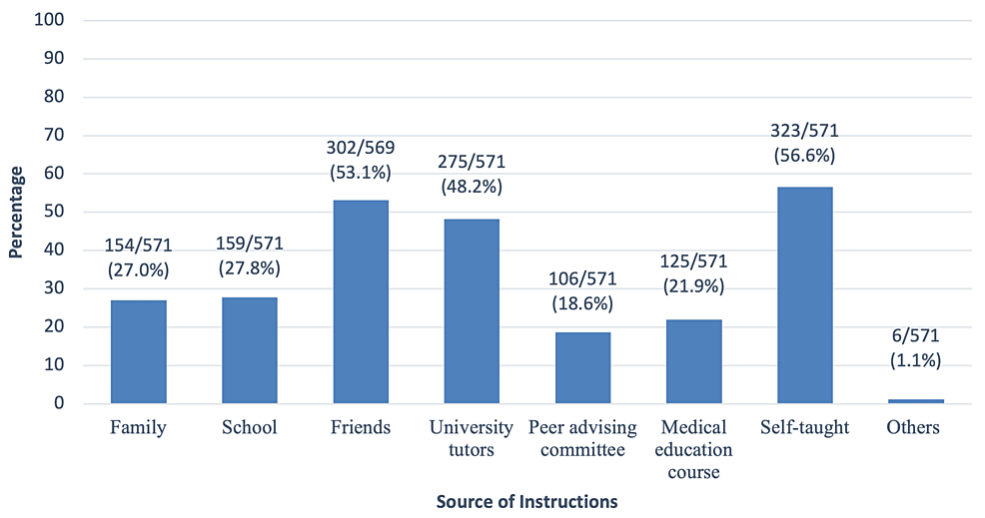
The source of instructions provided on different resources The data are represented above as frequency/valid total (valid percent). A valid total excludes missing data and accounts only for valid responses.

Medical students who used different resources for their academic studies were further questioned on whether they had received formal instructions on how to appropriately utilize those resources. Students were further asked to specify their main sources of the instructions. Figure 3 shows the source of instructions provided on different study resources. The majority of students 323/571 (56.6%) were self-taught, 302/569 (53.1%) received instructions from their friends and 275/571 (48.2%) received instructions from university tutors. A low percentage of students 125/571 (21.9%) received instructions from the medical education course while 106/571 (18.6%) received instructions from the peer advising committee (PAC). PAC is a student-based advising committee in which academically well-achieving students are selected among a group of volunteers to become members and represent their academic year. PAC members help the younger batch by providing advice, support, and instructions on how to succeed in the upcoming academic year based solely on their collective experiences. 

Factors influencing students’ selection of study resources

Medical students were asked on a five-point Likert scale about their perspectives on how different factors might impact their choices of study resources. Students identified two main factors influencing their choices and these were time and schedule along with the quality of information offered. Table 3 presents findings about the students’ agreement level with different statements concerning their choice of study resources. A total of 501/572 (87.6%) of students agreed or strongly agreed that time and schedule affect their choice of study resources. In addition, 457/571 (80.0%) of students agreed or strongly agreed that the quality of the information offered affects their choice of study resources. On the other hand, the least three factors that influenced students’ choice of study resources were language, money affordability of physical textbooks, and ease of remembering. A small percentage of students 172/570 (30.2%) agreed or strongly agreed that language can be a barrier to choosing the type of study resource. The inability to afford physical textbooks because they cost a lot of money was agreed upon or strongly agreed upon by only 203/569 (35.6%) of students. A total of 235/572 (41.1%) of students agreed or strongly agreed that it is easier to remember lectures taught online than studied in books.

**Table 3:** Reasons underlying students’ preferences in the selection of study resources *The valid total excludes missing data and accounts only for valid responses.

Statement	*Valid Total Responses	Strongly agree n (valid %)	Agree n (valid %)	Neutral (not sure) n (valid %)	Disagree n (valid %)	Strongly disagree n (valid %)
It's easier to remember the lecture taught online than studied in books.	572	110 (19.2)	125 (21.9)	208 (36.4)	94 (16.4)	35 (6.1)
My self-motivation affects my choice of study resources.	572	240 (42.0)	238 (41.6)	68 (11.9)	18 (3.1)	8 (1.4)
Time and schedule affect my study resources.	572	297 (51.9)	204 (35.7)	47 (8.2)	17 (3.0)	7 (1.2)
Language can be a barrier for choosing my study resources.	570	74 (13.0)	98 (17.2)	142 (24.9)	143 (25.1)	113 (19.8)
I can’t afford physical textbooks because they cost me a lot of money.	569	77 (13.5)	126 (22.1)	165 (29.0)	141 (24.8)	60 (10.5)
Peer pressure influence my choice of study resources.	567	90 (15.9)	178 (31.4)	144 (25.4)	89 (15.7)	66 (11.6)
I choose my study resources mainly based on the quality of information offered.	571	209 (36.6)	248 (43.4)	86 (15.1)	20 (3.5)	8 (1.4)
I have easy access to most of the digital resources provided by the university.	571	162 (28.4)	219 (38.4)	132 (23.1)	46 (8.1)	12 (2.1)
I have easy access to most of the physical resources provided by my university.	572	132 (23.1)	189 (33.0)	177 (30.9)	61 (10.7)	13 (2.3)
I am happy/satisfied with my academic performance.	572	113 (19.8)	196 (34.3)	160 (28.0)	64 (11.2)	39 (6.8)

Association between the frequency of using study sources by college and year of study 

Table 4 summarizes the association between different study resources and preclinical years by college and year of study. The data analysis of the relationship between the occasional use of textbook resources and the year of study indicated that the usage rate was around the same for first and second-year students 97(49.5%) and 94 (49.7%), respectively. However, the frequency of textbook usage decreased significantly amongst third-year students 66 (35.7%) as compared to that in students of previous years (P-value = 0.001). The type of college was found to have an association with the form of studying. More medicine students 198 (53.8%) were likely to choose recommended textbooks as opposed to 60 (29.4%) of dental medicine students (P-value <0.001). When considering the relationship between the college and the use of printed journals/articles, it was observed that both medical students and dental medicine students do not use printed journals/articles as a preferred study resource 254 (69.0%) and 135 (66.5%), respectively, (P-value = 0.625). Similarly, 223 (60.6%) medical students and 132 (64.7%) dental medicine students use printed lecture handouts every time or almost every time (P-value = 0.064). On the other hand, online website platforms were more utilized among students from the College of Medicine than by those in the College of Dental Medicine 171 (47.0%) compared to 53 (26.1%), respectively (P-value <0.001) (Table 4).

**Table 4:** Chi-square test results demonstrating the association between the frequency of using the different study resources by college and year of study *The valid total excludes missing data and accounts only for valid responses. A P-value less than 0.05 is considered statistically significant.

Use of Study Resources by College and Year of Study	*Valid Total	Never / Almost never n (valid %)	Occasionally / Sometimes n (valid %)	Every time / Almost every time n (valid %)	Chi-square value	P-value
Textbooks usage by year of study						
Year 1	570	57 (29.1)	97 (49.5)	42 (21.4)	17.794	0.001
Year 2		72 (38.1)	94 (49.7)	23 (12.2)		
Year 3		71 (38.4)	66 (35.7)	48 (25.9)		
Textbooks usage by college						
College of Medicine	572	83 (22.6)	198 (53.8)	87 (23.6)	71.712	<0.001
College of Dental Medicine		118 (57.8)	60 (29.4)	26 (12.7)		
Use of printed journals/articles by college						
College of Medicine	571	254 (69.0)	97 (26.4)	17 (4.6)	0.941	0.625
College of Dental Medicine		135 (66.5)	55 (27.1)	13 (6.4)		
Use of printed lecture handouts by college						
College of Medicine	572	75 (20.4)	70 (19.0)	223 (60.6)	5.495	0.064
College of Dental Medicine		26 (12.7)	46 (22.5)	132 (64.7)		
Use of online website platforms by college						
College of Medicine	567	64 (17.6)	129 (35.4)	171 (47.0)	35.214	<0.001
College of Dental Medicine		76 (37.4)	74 (36.5)	53 (26.1)		

## Discussion

Several studies have indicated that medical students commonly rely on digital and physical lecture handouts as their primary source of information [2,10,14]. Our research reinforces these findings and further highlights YouTube videos as the third most commonly used study resource among medical students. This observation is also consistent with the findings of the aforementioned studies. Interestingly, our study also indicates that recommended textbooks were among the least used resources, which is a deviation from the results observed in the latter studies.

According to Huon and colleagues, students typically prefer to study using digital lecture notes, recommended textbooks, and online quizzes [16]. Digital lecture handouts contain what is viewed as important concepts for the students to memorize while online quizzes focus on the most commonly tested concepts. On the other hand, recommended textbooks provide explanations on the rationale behind certain questions found in online quizzes as well as expanding on the concepts found in lecture handouts. This integrated approach to studying, in which multiple resources are used simultaneously, is important to maximize academic achievements. This supports Huon et al.'s suggestion that students’ resource selection and use is driven much more by assessment needs than by exploring for understanding [16].

Preclinical students concentrate more on the foundational concepts of anatomy, physiology, biochemistry, and other basic science subjects taught in medical school. These topics typically do not need to change much over short periods of time and are generally more stable than clinical subjects. Therefore, it is worth noting that when it comes to studying basic science subjects, textbooks are often the most reliable source of information. This is due to the rigorous review process that textbooks undergo prior to publication, which ensures their accuracy and comprehensiveness. Therefore, students and educators alike may find textbooks to be a more appropriate resource for these subjects. On the other hand, senior clinical students concentrate more on certain patient-centered care strategies and management plans which tend to be evolving due to emerging research. So, it is anticipated that as students progress in their academic years, particularly at the time of transition from preclinical to clinical years, they will begin to use fewer books, many of which are found to be outdated even before they're published [1] and thus, use online and printed journals or articles more often [13].

According to the literature, students read more articles as they advance in their academic years and get more research experience [13]. Being in the medical field, on the other hand, necessitates high search efficiency [1]. This means that students are expected to meet their study objectives within a short period. However, medical students face difficulties when studying using articles. This is because of the increased workload and decreased time available for them to accomplish their goals. As a result, an increasing number of students are turning to the use of search engines and educational applications that provide concise, to-the-point summaries of the required medical material because reading publications can be time-consuming.

Some of the sources that students use, including Wikipedia and WhatsApp, are questionable since the material they present is publicly available rather than coming from reputable organizations or individuals with training in research and medicine. Wikipedia, for instance, received the lowest ratings for both quality and dependability, as per Judd and Elliott’s study [7], even though a considerable number of students still utilize it. Thus, students should practice caution when using such resources. However, the increased use of WhatsApp in this study compared to other social media platforms may be due to the fact that it fosters communication among students, allows them to share resources outside of the classroom, connects them with medical professionals, and fosters their creativity [17].

Some university tutors, as well as groups designed to instruct and guide students, like medical education courses and PACs, strongly discourage using social media applications, general search engines, and unreliable websites like Wikipedia as study resources. Despite this discouragement, many students continue to use these study resources. Their ongoing use indicates a diminishing impact of these organizations on medical student’s resource preferences.

Nevertheless, when students were asked if they had received guidance on which sources to use in their preclinical years, their answers varied greatly, with self-taught, close friends and university professors taking the lead. This is a similar response to a study on the information-seeking behavior of medical students, who believed that a resource's reputation among peers and mentors was a crucial element in determining the veracity of information sources [12]. The same study concluded that in addition to the formal intentional information being provided by the university's medical education department on the use of resources specifically and many other aspects of university life generally, there is a hidden informal curriculum of advising preclinical students that functions through role modeling and older students' experiences and interactions with younger ones [12]. This conclusion also applies to the university in our study.

The selection of study resources is heavily influenced by accessibility, time efficiency, and the quality of the information. Digital resources are often thought to be easier to access than tangible resources like textbooks because the internet makes it simple to locate and access desired resources either at home or at work [1]. On the other hand, resources are considered to be of high quality when they are current and come from a reliable source [1]. These resources ought to be concise and to the point; they should not offer several unnecessary, time-consuming, in-depth explanations. According to students, summary sources are the finest of these [12]. Students will most likely select the source that strikes a balance between accuracy and accessibility.

Strength and novelty

Incorporating the entire preclinical medical student population spanning the first, second, and third years of both the College of Medicine and Dental Medicine enhances the authenticity of our results, bringing them into proximity with real-world data and consensus. Notably, this study is considered the first of its kind in the United Arab Emirates and is among the few studies conducted in the region, further underscoring its significance. Moreover, it lays the groundwork for subsequent research endeavors, offering a baseline for exploring the evolving dynamics as medical students progress in their academic journey, mature, and make more judicious use of resources. This longitudinal perspective facilitates result comparisons over time, providing insights into the evolving landscape of medical education.

Limitation and generalizability

The study's limitation arises from relying solely on quantitative methods, potentially limiting the depth of understanding. Integrating a mixed methods approach, including both quantitative and qualitative investigations, could have offered a more comprehensive exploration. Small group discussions and interviews would have enhanced understanding of the rationale behind students' study resource selection, facilitating a deeper analysis of the research topic. The University of Sharjah's medical students would, however, be a good representation of all medical students worldwide because the university was ranked among the best #121 universities in Asia and in rank #564 globally, and clinical medicine ranks #482 in the subject area [18,19].

Recommendation

The data used in this study was gathered before the implementation of online distance learning during the COVID-19 pandemic lockdown. Such an experience might change the students’ preferred choice of resources between physical and digital resources. These data may, however, be used to investigate whether the pandemic has popularized online learning or not.

## Conclusions

The incessant technological progressions that medical students witness as well as the diverse array of studying modalities introduce a constant challenge in discerning the most pertinent and practical approach to studying. Consequently, in response to these challenges, medical students endeavor to acquire a comprehensive knowledge base by integrating both physical and digital study resources. This strategic approach aims to optimize the efficacy and convenience of their studying methods, with resource selection guided by the quality of information provided and alignment with their individual time constraints and schedules. In addition, our study revealed that printed lecture handouts were the most commonly utilized physical study resource among medical students. In contrast, digital lecture handouts and YouTube videos emerge as the top two choices for digital resources. Conversely, social media, Wikipedia, and printed journals/articles rank as the least utilized resources. Remarkably, our findings clearly indicate that neither the medical education course nor the PAC emerged as primary sources of guidance for medical students in utilizing study resources. This observation underscores the imperative need for a continuous update of the curriculum within medical education courses to effectively address the evolving needs of the new generation of learners. Further research is warranted to establish correlations and identify which resources prove most pivotal in achieving academic success. This investigation holds the potential to provide valuable insights into refining educational strategies for medical students.
